# Steroid hormone signaling: multifaceted support of testicular function

**DOI:** 10.3389/fcell.2023.1339385

**Published:** 2024-01-05

**Authors:** Satoko Matsuyama, Tony DeFalco

**Affiliations:** ^1^ Reproductive Sciences Center, Division of Developmental Biology, Cincinnati Children’s Hospital Medical Center, Cincinnati, OH, United States; ^2^ Department of Pediatrics, University of Cincinnati College of Medicine, Cincinnati, OH, United States

**Keywords:** steroid, testis, hormone receptor, male fertility, sexual development, reproductive endocrinology

## Abstract

Embryonic development and adult physiology are dependent on the action of steroid hormones. In particular, the reproductive system is reliant on hormonal signaling to promote gonadal function and to ensure fertility. Here we will describe hormone receptor functions and their impacts on testicular function, focusing on a specific group of essential hormones: androgens, estrogens, progesterone, cortisol, and aldosterone. In addition to focusing on hormone receptor function and localization within the testis, we will highlight the effects of altered receptor signaling, including the consequences of reduced and excess signaling activity. These hormones act through various cellular pathways and receptor types, emphasizing the need for a multifaceted research approach to understand their critical roles in testicular function. Hormones exhibit intricate interactions with each other, as evidenced, for example, by the antagonistic effects of progesterone on mineralocorticoid receptors and cortisol’s impact on androgens. In light of research findings in the field demonstrating an intricate interplay between hormones, a systems biology approach is crucial for a nuanced understanding of this complex hormonal network. This review can serve as a resource for further investigation into hormonal support of male reproductive health.

## Introduction

Steroid hormones are integral to various physiological processes, including cellular metabolism, growth, immune function, and reproduction. It is essential to understand the complex biosynthetic pathways, site-specific production, and diverse actions of these hormones, in particular their roles in supporting the male reproductive system. Originating from cholesterol, these hormones undergo enzymatic transformations to form bioactive steroids, each with unique physiological roles.

The biosynthesis of steroid hormones, involving the conversion of cholesterol into a spectrum of hormones, is a crucial process. The review delves into the roles of steroid hormones in testicular function, emphasizing their interactions with intracellular nuclear receptors. Additionally, it explores the non-genomic signaling pathways of these hormones, highlighting their ability to elicit rapid cellular responses and showcasing their adaptability in cellular signaling (Cooke and Walker, 2022).

We will examine the roles of specific steroid hormones in the testes, such as androgens (including testosterone), estrogens, progesterone, cortisol, and aldosterone. We will also emphasize each hormone’s receptor function, localization within testicular cells, and the physiological impacts of their signaling. The significance of these hormones in male reproductive health is underscored by examining the effects of altered receptor signaling, addressing both deficiencies and excesses in hormonal actions.

By providing a broad overview of the roles of steroid hormones in the male reproductive system, we emphasize the need for a multidisciplinary and systems biology approach to fully understand the complex hormonal interactions in male reproductive health and their broader systemic implications.

## Biosynthetic pathway and site of production of steroid hormones

**FIGURE 1:**
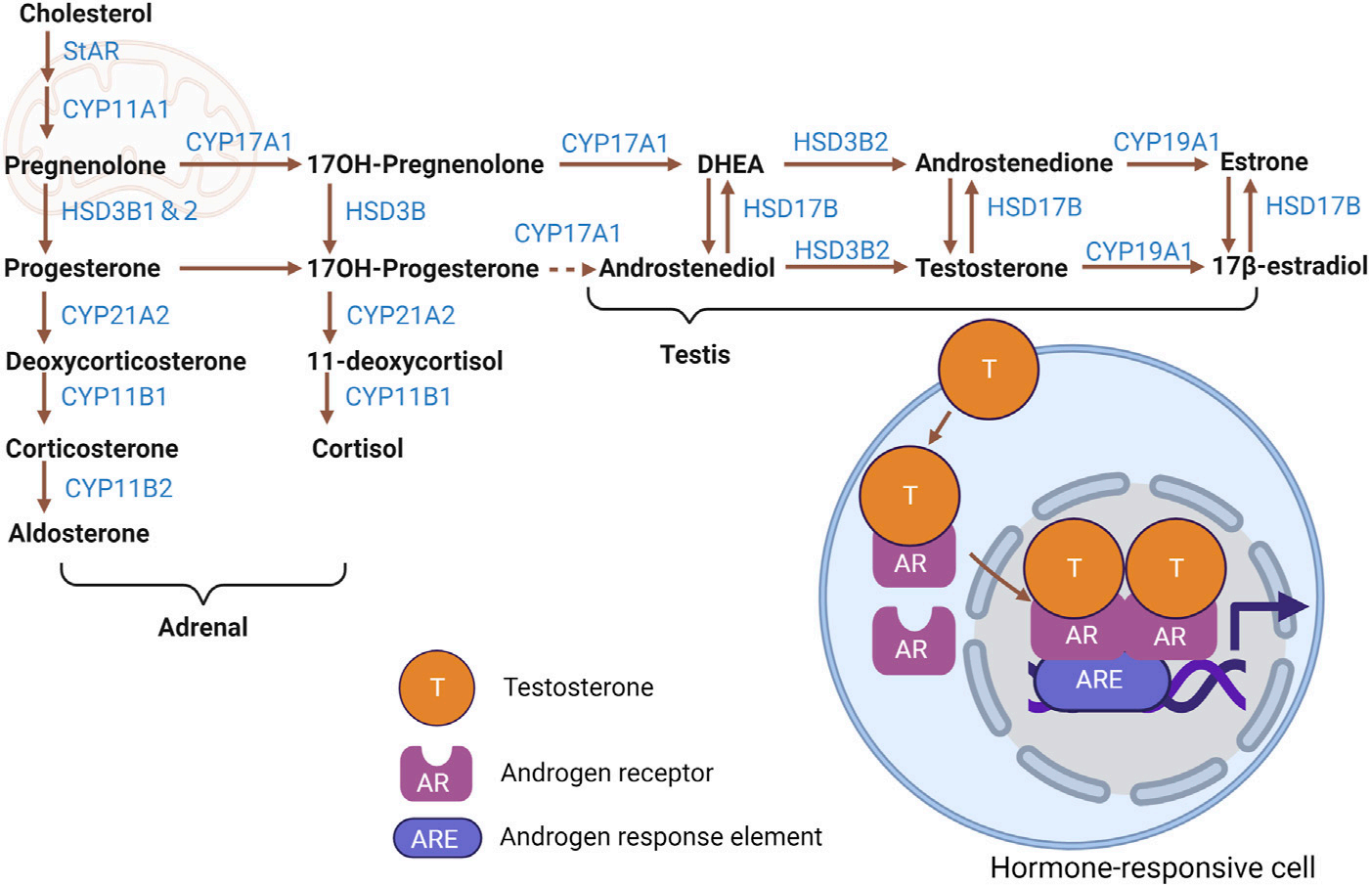
Steroid hormone biosynthetic pathways. Cartoon depicting biosynthesis of major adrenal- and testicular-derived steroid hormones from cholesterol, including intermediate molecules and steroidogenic enzymes involved. Testosterone and the resultant change in gene expression mediated by AR in a hormone-responsive cell is shown as an example of signaling activity. Figure created with BioRender.com.

The biosynthesis of steroid hormones (Figure 1) involves a cascade of biochemical reactions that transform cholesterol into various steroid hormones through the action of steroidogenic enzymes. Sources of intracellular cholesterol include free cholesterol, acetic acid, cholesterol esters found in lipid droplets, and cholesterol uptake from low-density lipoproteins (LDL) (Miller and Auchus, 2011; Miller, 2017; Zirkin and Papadopoulos, 2018).

Steroid hormones are immediately released into the blood after their synthesis, unlike peptide hormones that are stored in vesicles. This direct link between biosynthesis and release necessitates a readily available cholesterol pool in steroidogenic cells, since mitochondrial cholesterol, especially in the inner mitochondrial membrane where steroidogenesis starts, is insufficient for sustained hormone production. Recent research indicates that mitochondrial dynamics, autophagy, and associated lipophagy are vital for internal cholesterol uptake and balance in these cells, thereby supporting steroid hormone production essential for physiological functions (Bassi et al., 2021). In recent years, the role of mitochondria in steroid hormone action has been the focus of much attention (Guajardo-Correa et al., 2022; Picard and Shirihai, 2022; Shaia et al., 2023).

Within the mitochondria, the transport and conversion of cholesterol are facilitated by steroidogenic acute regulatory protein (StAR) and cytochrome P450 family 11 subfamily a member 1 (CYP11A1)—the latter being a key enzyme located on the inner mitochondrial membrane responsible for side-chain cleavage. Furthermore, the biosynthetic progression of steroid hormones from pregnenolone necessitates the involvement of cytochrome P450 (CYP) and hydroxysteroid dehydrogenase (HSD) enzymes, which are present in both mitochondria and the endoplasmic reticulum (Miller and Auchus, 2011; Zirkin and Papadopoulos, 2018).

Specifically, 3-beta-hydroxysteroid dehydrogenase (3β-HSD, official name HSD3B1) converts pregnenolone into progesterone. Progesterone is then metabolized into testosterone through the catalytic action of cytochrome P450 17A1 (CYP17A1), among other enzymatic steps. Testosterone undergoes conversion to estradiol by the enzyme aromatase, encoded by cytochrome P450 Family 19 subfamily a member 1 (CYP19A1). Cytochrome P450 family 21 Subfamily A member 2 (CYP21A2) mediates progesterone’s transformation to 11-deoxycortisol and deoxycorticosterone (DOC). After that transformation, cytochrome P450 family 11 subfamily B member 1 (CYP11B1) converts 11-deoxycortisol and DOC to the glucocorticoids cortisol and corticosterone, respectively. Finally, corticosterone is metabolized to the potent mineralocorticoid aldosterone by cytochrome P450 family 11 subfamily B member 2 (CYP11B2) (Miller and Auchus, 2011; Chakraborty et al., 2021).

Sex steroid hormones, primarily estrogens (estradiol, estriol, estrone), androgens, and progesterone, are predominantly synthesized in the gonads and placenta (Miller and Auchus, 2011). While the adrenal cortex secretes sex hormones, it does so in significantly lesser quantities than gonads. Interestingly, the gonads can also produce adrenal steroids (Chakraborty et al., 2021). In contrast, the adrenal cortex predominantly releases adrenal steroids, comprising glucocorticoids and mineralocorticoids (Miller and Auchus, 2011). In addition to steroid production by mesenchymal-derived Leydig cells in the testis, which are presumed to be the major steroidogenic cells in that organ, the importance of interstitial and peritubular macrophages in testicular steroidogenesis has also been discussed in recent years (Gu et al., 2022).

Feedback mechanisms finely tune steroid hormone biosynthesis. Elevated blood cortisol levels trigger a suppression of adrenocorticotropic hormone (ACTH) secretion from the hypothalamus and pituitary, resulting in a consequent decrease in cortisol release from the adrenal cortex (Schiffer et al., 2019). Other modulatory hormones include luteinizing hormone (LH) and follicle-stimulating hormone (FSH), which orchestrate hormone synthesis within the gonads (Schiffer et al., 2019). Regulation of testicular steroidogenesis is similarly complex, involving a diversity of critical regulatory pathways, including endocrine, paracrine, autocrine, and juxtacrine mechanisms (Bornstein et al., 2004; Hofmann and McBeath, 2022; Huhtaniemi and Toppari, 1995; O'Hara et al., 2015).

Testis-derived androgens and anti-mullerian hormone (AMH) drive male genital (internal and external) differentiation, while their absence leads to female differentiation. Testicular growth, testicular descent, and penile growth occur in response to testicular signals, with early infancy marked by active gonadotropin and steroid secretion. Reduced androgen signaling, characterized by hypogonadism, can inhibit male-specific sexual differentiation, such as penile growth, in boys. The reactivation of the HPG axis during puberty leads to secondary sex characteristic development, with hypogonadism potentially causing incomplete puberty and, later, infertility or sexual dysfunction (Grinspon et al., 2019).

## Mechanism of action of steroids in cells via intracellular nuclear receptors

Steroid hormones primarily exert their effects via nuclear signaling pathways (Chan and O'Malley, 1976a). In this cascade, steroid hormones traverse the cell membrane and subsequently associate with specific receptors in the cytoplasm and nucleus (Chan and O'Malley, 1976a). Acting as ligand-activated transcription factors, these receptors form complexes with distinct steroid hormones, such as androgens and estrogens (Chan and O'Malley, 1976b). Once a receptor-ligand complex is established, it interacts with a specialized region on the target gene’s regulatory region known as the hormone response element. The ligand-activated receptor dimer then binds specifically to this hormone response element (Devin-Leclerc et al., 1998). This binding prompts the recruitment of co-regulators to enhance gene transcription or co-repressors to inhibit it (Klinge, 2000). Resultant mRNA is then translated into protein, which culminates in characteristic hormonal responses, which include alterations in cell metabolism, growth, or differentiation.

Additionally, certain hormones can elicit rapid cellular responses in mere seconds upon administration, a phenomenon termed membrane or non-genomic signaling (Szego and Davis, 1967; Pietras and Szego, 1975). Interestingly, this non-classical signaling pathway can also elicit genomic effects (Fix et al., 2004).

## Androgens

### Receptor function

Androgens are essential for testicular function, male reproductive tract differentiation, and maintenance of male fertility (Cunha, 1973; Lasnitzki and Mizuno, 1977; Cunha et al., 1987). Androgens, most commonly testosterone, are ligands for androgen receptor (AR, also known as NR3C4). Androgen signaling is not strictly required for fetal testicular differentiation, as XY humans and mice can undergo fetal testicular differentiation without functional AR (Merlet et al., 2007). Nonetheless, androgens are absolutely essential for spermatogenesis and establishing male secondary sexual characteristics (De Gendt et al., 2004; Davey and Grossmann, 2016).

### Receptor localization

Even though androgens are required for spermatogenesis, germ cells do not express a functional androgen receptor (De Gendt et al., 2004), and AR is not required in germ cells in a normal somatic environment (Johnston et al., 2001). Therefore, androgen signaling acts through somatic cells to regulate sperm production. The persistent expression of AR in peritubular and Leydig cells starts from early fetal life and extends well into postnatal stages. In Sertoli cells, nuclear AR expression is first observed by 4–5 days after birth in mice, reaches high expression by 7–9 days of age, and is maintained in adult mice (Edelsztein et al., 2018). AR is expressed strongly in Sertoli cell nuclei but not in spermatogonia, preleptotene and pachytene spermatocytes, or round spermatids (Takada et al., 2023). Nuclear AR localization is linked to active signaling, whereas cytoplasmically-localized AR is considered inactive (Smith and Walker, 2014). Androgens cross the cell membrane and bind to AR in target cells, displacing heat shock proteins (HSP) (Shang et al., 2002). In the classical pathway, ligand-bound AR translocates to the nucleus and forms homodimers that interact with androgen response elements (ARE) in target gene promoters or with other transcription factors (TF), ultimately regulating gene expression (Smith and Walker, 2014). In the non-genomic pathway, the ligand-bound AR migrates to the inner side of the cell membrane, interacts with steroid receptor coactivator (Src), and activates the epidermal growth factor receptor (EGFR) signaling cascade involving downstream pathways such as the mitogen-activated protein kinase (MAPK), extracellular signal-regulated kinase (ERK), and cAMP response element binding protein (CREB) pathways (Konoplya and Popoff, 1992; Benten et al., 1999).

### Effects of loss of receptor signaling

Androgen signaling is vital for sperm development in the testis. Specifically, lack of testosterone or its receptor, AR, leads to infertility due to arrest in meiosis, a critical stage in spermatogenesis (Tsai et al., 2006; Walker and Cooke, 2023). This effect is predominantly mediated through Sertoli cells (Chang et al., 2004; Larose et al., 2020). Complete AR knockout (ARKO) in mouse models significantly reduces Sertoli cell numbers (Tan et al., 2005). Milder changes occur in Sertoli cell-selective AR knockout (SCARKO) mice, where meiosis is arrested at specific stages (Tan et al., 2005; Tsai et al., 2006). SCARKO mice also show a disrupted blood testis barrier, which is vital for spermatogenesis (Willems et al., 2010). Direct AR action on Sertoli cells is essential for developing spermatogonia and spermatocytes (O'Shaughnessy et al., 2010). An inducible AR knockout model (iARKO) was developed using a mouse line that ubiquitously expresses a tamoxifen-inducible Cre recombinase (Willems et al., 2011), in which AR inactivation can be temporally controlled, a feature that is lacking in ARKO and SCARKO mice. Both ARKO and iARKO mice exhibit reduced sperm production (Yeh et al., 2002; Willems et al., 2011).

Androgen action via testicular peritubular myoid cells is also essential for normal testis function, spermatogenesis, and male fertility, as well as for normal differentiation and function of adult Leydig cells (Welsh et al., 2012). Sertoli cell function was impaired in peritubular myoid-specific AR-knockout (PTM-ARKO) males, manifested by reduced seminiferous tubule fluid production and reduced expression of some androgen-dependent Sertoli-specific genes (Welsh et al., 2012). Functional AR in Leydig cells is required for steroidogenic function, as spermatogenic arrest predominately at the round spermatid stage was observed when *anti-Müllerian hormone receptor-2* (*Amhr2*) promoter-driven Cre was used to conditionally delete *Ar* in Leydig cells (Xu et al., 2007).

### Effects of excess receptor signaling

Prenatal exposure of male rat fetuses to excess testosterone disrupted reproductive function, manifested as a reduction of a number of parameters: testis weight; number of Sertoli cells, spermatocytes, and spermatids; sperm count and motility; and serum concentration of testosterone after puberty (Ramezani Tehrani et al., 2013). Letrozole, an aromatase inhibitor, causes estrogen deficiency and androgen excess. Severe testicular defects, including necrosis and disruption of the seminiferous epithelium, sloughing of epithelial cells, and spermatogenic arrest were observed in prenatal letrozole-treated groups (Shaaban et al., 2023), demonstrating the negative impacts of excess androgens. In the SPARKI (SPecificity-affecting AR KnockIn) mouse model, in which a zinc finger of the AR protein was replaced with that of GR such that only classical, and not selective, androgen response elements can be bound, reproductive capacity and organ size were significantly compromised (Schauwaers et al., 2007).

## Estrogens

### Receptor function

Two primary pathways dictate estrogen signaling. The classical pathway involves the nuclear estrogen receptors ERα (ESR1) or ERβ (ESR2) (King and Greene, 1984; Green et al., 1986; Greene et al., 1986; Kuiper et al., 1996). These receptors engage with estrogen response elements (EREs) in the DNA to regulate gene expression (Edwards, 2005). The non-classical pathway involves GPER/GPR30, a G protein-coupled receptor, which triggers rapid signaling events such as cAMP production and calcium release upon binding with estrogen (Filardo et al., 2000; Filardo et al., 2002; Revankar et al., 2005). Within the testis, GPER is found on Sertoli cells (Lucas et al., 2010) and sperm (Liu et al., 2009), influencing sperm activation and movement via estrogen’s non-genomic effects.

### Receptor localization

Numerous studies have reported the presence of ERα in peritubular cells, Leydig cells (Fisher et al., 1997; Pelletier et al., 2000; Mäkinen et al., 2001), and, notably, Sertoli cells (Saunders et al., 2001; Cavaco et al., 2009; Valeri et al., 2020). However, the expression dynamics of ERα in mouse testes show a decline from mid to late puberty (Jefferson et al., 2000). A consistent pattern has been observed in human samples, showing ERα expression in Sertoli cells from childhood through puberty (Valeri et al., 2020). In contrast, ERβ expression in mouse testes reaches its zenith on postnatal days 1–5 and is seldom seen after postnatal day 12 (Cooke et al., 2017). In adult mice, ERβ is found in Leydig and germ cells but is conspicuously absent from Sertoli cells (Cavaco et al., 2009). The most prominent expression was observed in the nucleus of pachytene spermatocytes, but ER expression was also evident in spermatogonia, preleptotene spermatocytes, round spermatids, and Sertoli cells (Takada et al., 2023).

### Effects of loss of receptor signaling

Estrogen receptor anomalies stemming from mutations in *Esr1* (encoding ERα) and *Esr2* (encoding ERβ) lead to estrogen insensitivity or resistance conditions. In *Esr1*-deficient mice, fetal testis development is unaffected, but in adulthood they exhibit disrupted spermatogenesis and excess fluid retention in the rete testis, leading to infertility (Lubahn et al., 1993). Also, loss of ER function in ERKO males leads to reduced mating frequency, low sperm numbers, and defective sperm function (Eddy et al., 1996). Patients with aromatase deficiency exhibit symptoms such as Sertoli cell hyperplasia and significant testicular enlargement, likely due to continuous FSH elevation (Morishima et al., 1995). Intriguingly, murine studies suggest ERβ′s minimal role in testicular physiology (Krege et al., 1998; Dupont et al., 2000). An interesting relationship between the decline in endogenous estrogen and testicular cortisol has been observed, hinting at an interconnected signaling pathway impacting Sertoli cell proliferation. For instance, treatment with the aromatase inhibitor letrozole led to a reduction in testicular cortisol levels, inversely increasing Sertoli cell proliferation (Berger et al., 2019).

### Effects of excess receptor signaling

A rare genetic aberration in humans, termed aromatase excess syndrome, results from a chromosome 15 rearrangement that causes an upsurge in CYP19A1 expression (Fukami and Ogata, 2022). Remarkably, male external genitalia and fertility remain unaffected. This suggests elevated estrogen levels do not significantly hamper testicular function (Rochira and Carani, 2009; Bulun, 2014; Miedlich et al., 2016). Following chronic treatment with estradiol, however, a reduced number of germ cells was observed, likely caused by increased germ cell apoptosis (Kaushik et al., 2010b). All these observations were attributed to the negative effect of estradiol on the expression of AR, as estrogen treatment causes an increase in ERα expression and a decrease in AR expression in the rat testis (Kaushik et al., 2010a; b). Analysis of human testicular morphology and function after estradiol treatment revealed decreased seminiferous tubule diameter and induced fatty degeneration in the surrounding connective tissue. Spermatogenesis was impaired, resulting in mainly spermatogonia being present (Leavy et al., 2017). Genetically induced estrogen receptor α mRNA (*Esr1*) overexpression did not adversely affect fertility development in male mice (Heath et al., 2011), revealing a minimal impact of excess estrogen signaling on general testicular function in the absence of an exogenous estrogen exposure.

## Progesterone

### Receptor function

Traditionally associated with female reproductive physiology for its roles in fertilization, pregnancy, and endometrial receptivity, progesterone also exerts important functions in male reproductive tissues like the prostate and testes. Progesterone rapidly activates intracellular signaling in human sperm, regulating key aspects of their physiology (Publicover and Barratt, 2011). An ion channel unique to the sperm tail seems to relay progesterone’s signal (Lishko et al., 2011; Strünker et al., 2011), although it is unclear if progesterone signals through the progesterone receptor (PR; official name PGR) in the testis (Baker and Katsu, 2020).

### Receptor localization

Experiments focusing on suppressing spermatogenesis and its effects on receptor expression found that PR-B, a specific isoform of the progesterone receptor, was expressed in the rat testis at both transcriptional and protein levels (Lue et al., 2013). In mice, B-gal expression from a PR knockout allele was reported to be expressed in PRKO mice within Leydig cells, but only under conditions of gonadotropin inhibition (Lue et al., 2013); however, there are caveats in relying on a reporter line, and immunohistochemistry was not performed. There is also some debate about PR expression in primate and human testis, in which some studies report widespread testicular PR expression (Shah et al., 2005), while others show limited expression in few cells (Luetjens et al., 2006). Given some inconsistency between different reports, the localization of PR in the testis, if any is indeed expressed, is unclear.

### Effects of loss of receptor signaling

Mice devoid of functional progesterone receptor (PRKO) had significantly larger testes than control mice, and they exhibited increased sperm production accompanied by an increase in Sertoli and Leydig cell numbers (Lue et al., 2013). In general, it appears that PR plays an inhibitory role in testicular function.

### Effects of excess receptor signaling

Progesterone treatment suppresses gonadotropin-releasing hormone (GnRH) in the hypothalamus, which in turn lowers the production of LH and FSH by the pituitary gland (Amory, 2004). Synthetic progestins such as levonorgestrel induced germ cell apoptosis (Lue et al., 2013); therefore, progesterone may also act directly on the testes, but the mechanism has not yet been elucidated. Testicular macrophages could also produce progesterone, and steroid production by macrophages may contribute to a local feedback loop between Leydig cells and macrophages that regulate testosterone production (Yamauchi et al., 2022; Ogawa and Isaji, 2023). This interplay suggests that testosterone and progesterone can influence each other’s roles in testicular function.

## Cortisol

### Receptor function

Glucocorticoids regulate major systemic functions, including blood pressure and immune responses. Corticosterone is the major form in mice (Leenaars et al., 2020), whereas it is cortisol in humans (Salehzadeh and Soma, 2021). The primary signaling mechanism involves the glucocorticoid receptor (GR; official name NR3C1), a nuclear receptor family member. GR modulates gene expression when bound to cortisol by interacting with specific DNA elements (Oakley and Cidlowski, 2013). Subcellular trafficking, promoter specificity, cofactor interaction, receptor stability, and turnover further fine-tune the receptor’s cellular functions (Whirledge and Cidlowski, 2017). The response to glucocorticoids is regulated by the recruitment of cofactors, which can act in various mechanisms including remodeling of chromatin, facilitating the assembly of transcriptional machinery, or modifying histones or other components of the transcription factor complex (Barnes, 2009; Grøntved et al., 2013; Jones et al., 2014). The testicular role of GR is less well understood. Innate immunity, which is responsive to GR signaling, remains crucial despite the blood-testis barrier and systemic immune tolerance that shield the testis from inflammatory responses normally occurring in other organs. Inflammation can compromise this barrier and induce germ cell death, yet glucocorticoids’ role in testicular function is unclear (Whirledge and Cidlowski, 2017).

### Receptor localization

In the testis, the localization and role of GR are less extensively studied as compared to other tissues. GR expression was reported in Leydig, peritubular, Sertoli cells and early germ cells on postnatal day 20 testis in mice (Hazra et al., 2014). In adult mice, GR was expressed in the nuclei of spermatogonia and preleptotene spermatocytes. In contrast, pachytene spermatocytes exhibited weak GR expression, and no apparent GR expression was observed in spermatids (Takada et al., 2023). In mature 70-day-old mice, Sertoli cells do not express detectable levels of GR (Levy et al., 1989; Hazra et al., 2014).

In adult human testis, GR was detected in peritubular cells (Welter et al., 2020), a subset of Leydig cells, Sertoli cells (weak), and spermatogonia, but not in spermatids. The GR expression pattern in fetal testis samples differed, notably by heterogeneous expression in Sertoli cells, lack of expression in gonocytes, and weak expression in nascent peritubular cells, along with detectable expression in a subset of prospermatogonia (Nordkap et al., 2017).

### Effects of loss of receptor signaling

Sertoli-specific *Gr* deletion in mice had a limited impact on fertility but did reduce Sertoli cell and spermatocyte numbers (Hazra et al., 2014). A 50% reduction of GR in Leydig cells using AAV9-Cre disrupted steroidogenesis (Gannon et al., 2022), suggesting a role for GR in Leydig cell function. Rat adrenalectomy studies showed decreased sperm counts were partially restored by dexamethasone, but not sperm morphology (Silva et al., 2014). Testosterone production surged after adrenalectomy, which glucocorticoid replacement prevented (Whirledge and Cidlowski, 2017), revealing an interplay between glucocorticoid and testosterone levels. Mice deficient for *Tsc22d3-2*, originally identified as a dexamethasone-induced transcript protecting T lymphocytes from T cell receptor (TCR)/CD3-activated cell death (D'Adamio et al., 1997), exhibited increased germ cell apoptosis, leading to azoospermia and infertility (Romero et al., 2012; Suarez et al., 2012).

### Effects of excess receptor signaling

Glucocorticoids like betamethasone and dexamethasone disrupt male reproductive hormones across species. In medaka fish, betamethasone led to feminization (Su et al., 2023). Dexamethasone treatment in mice increased expression of male-specific genes like *Sry* and *Sox9* during critical embryonic stages (Yun et al., 2016). In rats treated with glucocorticoids during prenatal development, expression of nuclear COUP-TFII (official name NR2F2), a marker of Leydig precursors, increased, stem Leydig cell proliferation decreased, and testosterone levels decreased (van den Driesche et al., 2012); in addition, they showed compromised testicular morphology and reduced StAR expression (Liu et al., 2018). Epigenetic programming affecting sperm quality was also observed (Liu et al., 2023).

Postnatal exposure to excess glucocorticoids also has significant impacts. Psychological stress, which increases systemic glucocorticoid levels, impaired Sertoli cell function in adult rats (Medubi et al., 2021). Dexamethasone led to atrophy of the seminiferous tubules and degeneration of spermatocytes but was somewhat mitigated by onion extract, which has the potential to inhibit oxidative stress, in adult rats (Nassan et al., 2021). Following exposure, adult horses showed gene downregulation such as *NR4A1*, *NR5A1,* and *NR5A2* in primary cultures of Leydig cells, but *A. cepa* (onion) extract inhibited oxidative stress induced by dexamethasone (Valdez et al., 2019). In male rats, dexamethasone for 7 days caused severe testicular pathology such as hypospermatogenesis, germ cell degeneration and depletion, epithelial vacuolization, and degenerated Leydig cells (Azimi Zangabad et al., 2023).

*In vitro* studies indicate dexamethasone suppresses Leydig cell differentiation (Zhang et al., 2019; Liu et al., 2021), an effect reversible with an NR3C1 (GR) antagonist (Zhang et al., 2019). These findings highlight the need for further study on long-term consequences of glucocorticoid treatment (Jeje and Raji, 2017).

## Aldosterone

### Receptor function

Aldosterone regulates sodium and water homeostasis via the mineralocorticoid receptor (MR; official name NR3C2) and has broader roles in organ development, including the kidneys and heart (Zhang et al., 2018).

### Receptor localization

MR is expressed in the testicular Leydig cells of adult rats, where aldosterone modulates stem Leydig cell proliferation and testosterone production (Zhang et al., 2018).

### Effects of loss of receptor signaling

The consequences of MR knockout in testes are unclear. Fetal exposure to di-(2-ethylhexyl) phthalate (DEHP) reduced MR mRNA and protein levels and reduced testosterone levels in adulthood (Martinez-Arguelles et al., 2009), suggesting a link between MR and testicular steroidogenesis.

### Effects of excess receptor signaling

Low serum testosterone correlates with gonadal dysfunction. Similar observations in rats with renal failure suggest a link between the renin-angiotensin-aldosterone system and gonadal failure (Zhang et al., 2018; Liu et al., 2021). *In vitro* culture of seminiferous tubules from Leydig-cell-depleted testes revealed that aldosterone suppressed stem Leydig cell proliferation and increased steroidogenesis, which is in direct contrast with dexamethasone’s inhibitory effect on stem Leydig cell differentiation (Zhang et al., 2018).

Both aldosterone and progesterone activate their respective receptors at low concentrations, so the antagonistic effect of elevated progesterone on human MR needs further study (Zhang et al., 2018; Baker and Katsu, 2020). A human MR Ser810Leu mutation that is activated by progesterone is associated with high blood pressure (Geller et al., 2000). Furthermore, spironolactone, an aldosterone antagonist, is linked to reduced plasma testosterone in men (Baba et al., 1978). All these data indicate an interplay between aldosterone levels and the production of other steroid hormones.

## Future perspectives

Immune cells are not merely passive targets of steroid hormones through their receptors but also possess the potential to convert and metabolize these hormones on their own (Rubinow, 2018). Human alveolar macrophages have been reported to convert androstenedione to testosterone and other steroids through specific enzymatic catalytic activities (Milewich et al., 1983). Moreover, human monocyte-derived macrophages can convert dehydroepiandrosterone (DHEA) to testosterone, estradiol, and other steroids in the presence of LPS (Schmidt et al., 2000). These findings offer insights into the heterogeneity and microenvironment-dependency of steroid hormone conversion in macrophages.

Furthermore, it has been shown that immune cells have the capacity for *de novo* steroidogenesis starting from cholesterol. For instance, the expression of StAR has been detected in macrophages (Ma et al., 2007; Taylor et al., 2010), suggesting at least the ability to import cholesterol into mitochondria and to produce steroidogenic substrates. Primary rat testicular macrophages have been reported to produce corticosterone *in vitro* (Wang et al., 2017), although it remains unclear whether this production is derived from *de novo* steroidogenesis or from the conversion of other steroids. Furthermore, it has been reported that testicular macrophages produce progesterone *de novo*, which is promoted by cAMP and inhibited by M1 polarization inducers (Yamauchi et al., 2022). These discoveries suggest the potential involvement of macrophages in local feedback loops with Leydig cells, thus influencing and contributing to the regulation of testosterone production.

The existence of steroidogenesis and steroid signaling within immune cells presents new possibilities for understanding how immune cells communicate and shape the physiology of immune responses, as well as how they become dysregulated in disease states. The impact of nuclear receptor signaling pathways, such as those mediated by steroid hormone receptors, is profound. Given that the GR alone can regulate up to 20% of genes, these pathways hold the potential to revolutionize our understanding of immune regulation (Galon et al., 2002; Weikum et al., 2017). Technological advancements, particularly improvements in liquid chromatography/tandem mass spectrometry, single-cell transcriptomics, multimodal omics, and spatial resolution techniques, are enhancing our understanding of steroid production and gene expression networks related to steroid responsiveness in immune cells. Chromosome conformation capture assays can analyze changes in genome interactions, contributing to the elucidation of mechanisms by which nuclear receptors bind to chromatin and regulate gene expression (Chakraborty et al., 2021). These developments are poised to deepen our understanding of immune regulation and contribute to the development of new therapeutic strategies.

## Discussion

**FIGURE 2:**
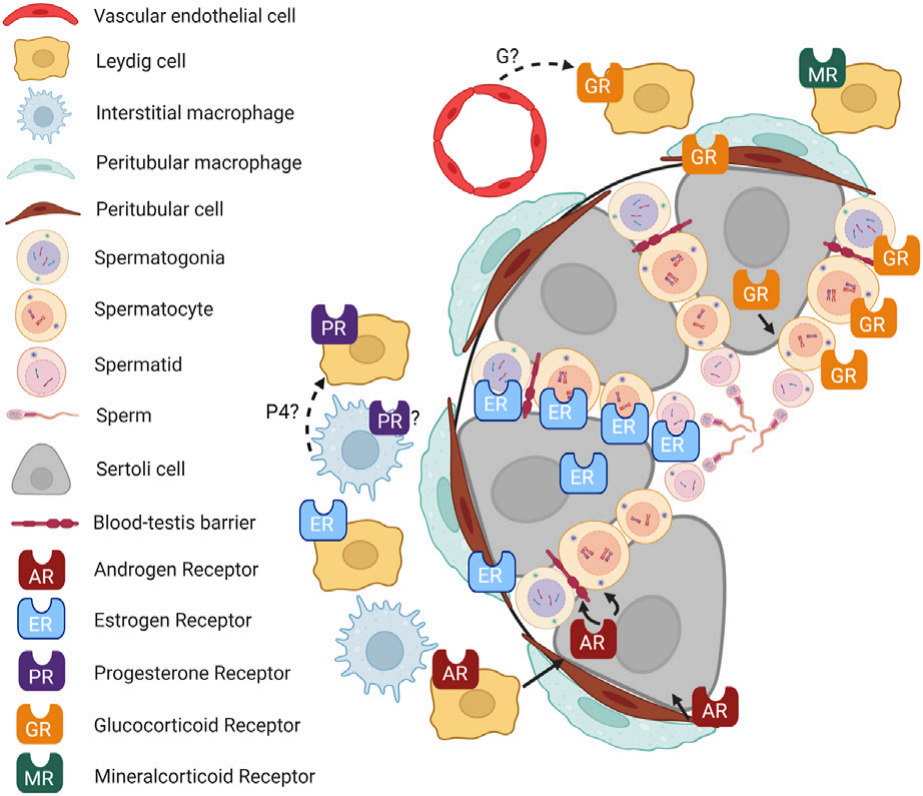
Hormone signaling in the testis. Cartoon depicting an adult mouse testis, showing localization of hormone receptors in different cell types and known (solid lines) and putative (dashed lines) interactions between cell types. P4, progesterone; G, glucocorticoid. Figure created with BioRender.com.

The multifaceted roles of hormone receptors in testicular function have been the subject of considerable research. Still, the complexity of their interactions and the mechanisms underlying their effects on male reproductive health remain incompletely understood (Figure 2). In particular, the roles of GR, MR, and PR are still unclear. Similarly, hormone receptor localization in testicular cells warrants further study.

Furthermore, hormones often do not function in isolation. Their role in the testes appears modulated by complex feedback loops, crosstalk with other hormones, and possibly interaction with local immune cells (Rubinow, 2018). As immune-mediated endocrinology gains traction in the scientific community, it would be informative to investigate how local immune cells like macrophages interact with testicular hormone production and action.

In addition, dysfunction in hormonal signaling is not without repercussions. Aberrant expression or activities of hormonal receptors can lead to a variety of pathologies, including inflammation-induced infertility, testicular atrophy, and even testicular cancer. Understanding these outcomes necessitates a multi-disciplinary approach that transcends traditional endocrinology.

In conclusion, a systems biology approach integrating these multifactorial interactions will likely provide new insights into male reproductive endocrinology. Such holistic perspectives are imperative not just for solving reproductive health issues but also for advancing our understanding of systemic endocrine functions.
